# Increase of miR-199a-5p by protoporphyrin IX, a photocatalyzer, directly inhibits E2F3, sensitizing mesenchymal tumor cells to anti-cancer agents

**DOI:** 10.18632/oncotarget.2928

**Published:** 2015-02-24

**Authors:** Jung Min Lee, Mi Jeong Heo, Chan Gyu Lee, Yoon Mee Yang, Sang Geon Kim

**Affiliations:** ^1^ College of Pharmacy and Research Institute of Pharmaceutical Sciences, Seoul National University, Seoul 151-742, Republic of Korea

**Keywords:** E2F3, protoporphyrin IX, miR-199a-5p, chemotherapeutic agent, hepatocellular carcinoma

## Abstract

Hepatocellular carcinoma (HCC) is a leading cause of cancer-related deaths. Protoporphyrin IX (PPIX) has been used for photodynamic therapy. Mesenchymal cancer cells adapt to tumor microenvironments for growth and metastasis possibly in association with miRNA dysregulation. In view of the effect of PPIX on cancer-related genes, and its potential to inhibit tumor growth and migration/invasion, this study investigated whether PPIX enables mesenchymal liver tumor to restore dysregulated miRNAs, and if so, whether it sensitizes the cancer cells to chemotherapy. In addition, we explored new target(s) of the miRNA(s) that contribute to the anti-cancer effects. Of the ten miRNAs predicted by the 3′-UTR of HIF-1α mRNA, PPIX treatment increased miR-199a-5p, leading to the inhibition of E2F3 expression which is upregulated in mesenchymal liver tumor. miR-199a-5p levels were downregulated in HCC with E2F3 overexpression. An approach modulating epithelial-mesenchymal transition provided the expected changes in miR-199a-5p and E2F3 *in vivo*. PPIX prevented tumor cell growth and migration/invasion, and had a synergistic anti-cancer effect when combined with chemotherapeutics. In a xenograft model, PPIX treatment decreased overall growth and average tumor volume, which paralleled E2F3 inhibition. Overall, PPIX inhibited growth advantage and migratory ability of cancer cells and sensitized mesenchymal liver tumor cells to chemotherapeutics.

## INTRODUCTION

Aggressive tumor cells are often resistant to chemotherapeutic agents and may undergo the epithelial-mesenchymal transition (EMT) process through increases of zinc finger E-box binding homeobox 1/2 (Zeb1/2), snail, and twist, and the loss of E-cadherin [1]. In addition, EMT promotes cancer cell migration, invasion, and metastasis [2]. Several porphyrin-based chemicals have been approved for the treatment of certain cancers (Photofrin^®^ and Foscan^®^) or actinic keratosis (Levulan^®^ and Metvix^®^) due to their light-absorbing properties [3, 4]. Silencing of ferrochelatase, an enzyme responsible for the last step of heme biosynthesis, promoted 5-aminolevulinic acid (ALA)-induced photodynamic anti-cancer effect by increasing intracellular protoporphyrin IX (PPIX) content [5]. In a previous study, we found that PPIX has an anti-tumor effect in colon cancer cells. However, it was unclear whether PPIX has an effect against tumor cells with the EMT phenotype. In the present study, we investigated whether PPIX treatment antagonizes the proteins specifically overexpressed during the process of EMT, and if so, whether PPIX sensitizes tumor cells to chemotherapy.

MicroRNAs (miRNAs) bind with the 3′-untranslated region (3′-UTR) of complementary target mRNAs and cause degradation and/or translational repression [6]. Dysregulation of miRNAs is a common feature of malignant tumors, and may function to promote oncogene activation and/or downregulate tumor suppressors [7, 8]. A specific miRNA expression profile is developmentally controlled and is changed with differentiation in different cell types. Aberrant expression of miRNAs may contribute to tumor cell proliferation and migration/invasion [7, 9]. Previously, we found that PPIX suppresses HIF-1α by inhibiting the chaperone activity of HSP90 [10]. We also observed that PPIX treatment with cobalt chloride decreased HIF-1α level at time zero in an experiment using cycloheximide, indicative of an additional inhibition of *de novo* protein synthesis. Since HIF-1α levels are controlled by transcriptional and translational mechanisms [11], we wondered whether the effect of PPIX on HIF-1α could also result from post-transcriptional alterations by miRNAs.

Microenvironments where a supply of oxygen and nutrients are considerably limited play a crucial role in angiogenesis and cancer cell migration/invasion [12, 13]. The E2F transcriptional factors are the downstream targets of a certain tumor suppressor (i.e., retinoblastoma gene), playing a role in cell proliferation, apoptosis, differentiation, and tumor development [14, 15]. Since aggressive functions of cancer cells with phenotypic changes are orchestrated by various molecules and signaling pathways in the need of adaptation to tumor microenvironments, we were interested in other molecules regulated in conjunction with HIF-1α accumulation, and found that E2F3 expression was also controlled by common miRNAs interacting with the HIF-1α mRNA.

Of the miRNAs putatively interacting with the HIF-1α mRNA, we narrowed our focus to the effect of PPIX on miR-199a-5p because the particular miRNA regulates cancer-related genes and its expression levels decrease in various cancers including liver, colon, breast, bladder, and testicular cancers [16–19]. In addition, treatment of cancers with miR-199a-5p mimic enhances chemosensitivity by regulating autophagy [17, 20]. We discovered that PPIX treatment markedly increased miR-199a-5p levels in tumor cells, and this effect resulted in the inhibition of E2F3, a key regulator of G1/S transition and tumor growth [21]. Moreover, our results obtained from cell-based studies and/or animal experiments indicate that PPIX sensitizes a mesenchymal type of cancer cells to chemotherapeutic agents so that combinatorial treatments of PPIX with representative chemotherapeutics may synergistically inhibit growth advantage and migrating capability of malignant liver tumor cells.

## RESULTS

### HIF-1α overexpression in mesenchymal HCC cells and chemosensitization by PPIX

**Figure 1:**
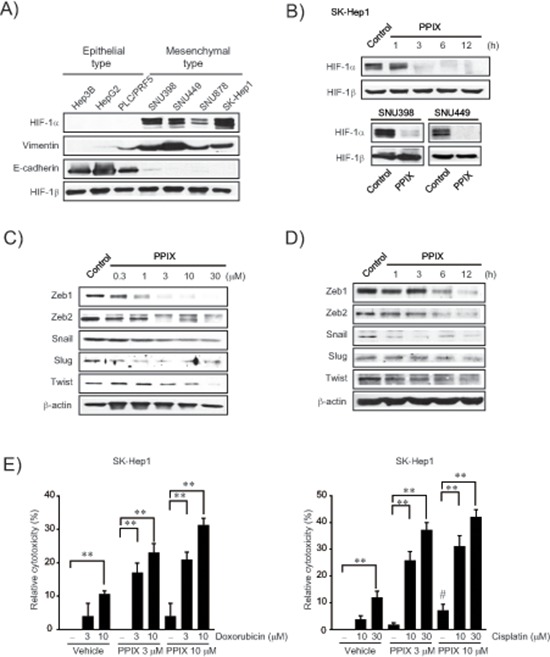
Inhibition of EMT markers by PPIX in mesenchymal cancer cell lines **(A)** HIF-1α expression and EMT markers in liver tumor cell lines. **(B)** Inhibition of HIF-1α by PPIX treatments. SK-Hep1 cells were incubated in a medium containing 3 μM PPIX for 1–12 h, whereas SNU398 and SNU449 cells were treated with 3 μM PPIX for 3 h. Equal loading of proteins was verified by immunoblottings for HIF-1β. **(C)** The concentration-response of PPIX in SK-Hep1 cells. The cells were incubated with the indicated concentrations of PPIX for 3 h. **(D)** The time-course effects of PPIX. SK-Hep1 cells were treated with 3 μM PPIX for 1–12 h. Equal loading of proteins was confirmed by immunoblottings for β-actin. **(E)** Chemosensitization of mesenchymal liver tumor cells by PPIX. SK-Hep1 cells were exposed to PPIX (3 or 10 μM) with or without the indicated concentrations of doxorubicin (left) or cisplatin (right) for 48 h. Value represents the mean ± S.E. from four independent experiments (treatment mean significantly different from vehicle- or PPIX-treated group, **P < 0.01, or vehicle group, #*P* < 0.05).

To confirm the relationship between EMT and HIF-1α, we measured basal HIF-1α expression in a series of liver tumor cell lines, and found that HIF-1α levels were greater in cancer cells with mesenchymal phenotype (i.e., SNU398, SNU449, SNU878, and SK-Hep1) than in those with epithelial phenotype (i.e., Hep3B, HepG2, and PLC/PRF5) (Figure 1A). Mesenchymal characteristics were verified by vimentin upregulation as well as E-cadherin repression (PLC/PRF-5 is classified as an epithelial cell type despite slight expression of vimentin [22]). Of note, PPIX treatment (3 μM) almost completely inhibited HIF-1α overexpressed in SK-Hep1, SNU398, and SNU449 cells (Figure 1B). Moreover, PPIX downregulated Zeb1/2, snail, slug, and twist levels in SK-Hep1 cells in a concentration- and time-dependent manner (Figure 1C and 1D), consistent with the finding that hypoxia facilitates EMT with HIF-1α overexpression [23, 24].

To determine whether PPIX treatment chemosensitizes mesenchymal liver tumor cells to anti-cancer agents, we next assessed the effect of PPIX alone or in combination with chemotherapeutic agents on the cytotoxicity of a representative mesenchymal liver tumor cell. In this experiment, we used doxorubicin and cisplatin because these agents alone or in combination with others have been widely applied for cancer chemotherapy but elicit chemoresistance through miRNA dysregulation [20, 25, 26]. Although PPIX treatment alone was moderately cytotoxic to SK-Hep1, a combinatorial treatment of PPIX with either doxorubicin or cisplatin synergistically enhanced cytotoxic activities as compared to each treatment alone (Figure 1E). Our results indicate that PPIX has a cytotoxic and chemosensitizing effect on mesenchymal liver tumor cell.

### Upregulation of miR-199a-5p by PPIX

**Figure 2:**
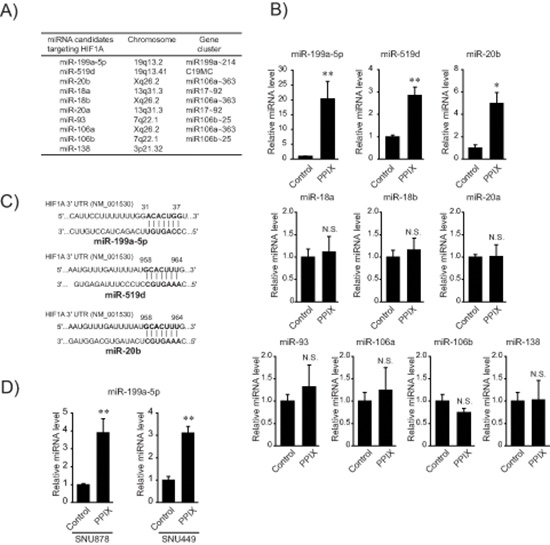
Induction of miR-199a-5p by PPIX **(A)** A list of miRNA candidates targeting *HIF1A*. The miRNAs were found using TargetScan 6.1 and miRanda. **(B)** The effect of PPIX on the expression of miRNAs targeting *HIF1A*. The levels of miRNAs were determined by qRT-PCR assays in SK-Hep1 cells treated with 3 μM PPIX. **(C)** Sequence motifs of the 3′-UTR of *HIF1A* match seed sequences of candidate miRNAs. **(D)** The levels of miR-199a-5p after PPIX treatment in mesenchymal HCC cells. qRT-PCR assays for miR-199a-5p transcripts in SNU878 or SNU449 cells. For B and D, the data represent the mean ± S.E. from 3 or 4 different independent experiments (significantly different from vehicle-treated control, *P < 0.05; **P < 0.01; N.S., not significant).

Since miRNAs orchestrate post-transcriptional regulation of HIF-1α, we were interested in the effect of PPIX on the expression of miRNAs that interact with the 3′-UTR region of HIF-1α mRNA. Analysis of TargetScan 6.1 database and miRanda enabled us to extract the known or putative miRNAs predicted to bind to the mRNA (Figure 2A). Of the miRNAs extracted using computer algorithms, PPIX treatment substantially increased miR-199a-5p levels and to moderate degrees those of miR-519d and -20b in SK-Hep1 cells (Figure 2B and 2C). Since the rest of the miRNAs were unchanged in subsequent experiments, we narrowed our focus to miR-199a-5p, a liver-enriched miRNA, because fold-increase of the miRNA was the greatest and basal expression of the other miRNAs was relatively low. We also confirmed the ability of PPIX to increase miR-199a-5p in other mesenchymal tumor cells, SNU878 and SNU449 (Figure 2D).

### Identification of E2F3 as a new target of miR-199a-5p

**Figure 3:**
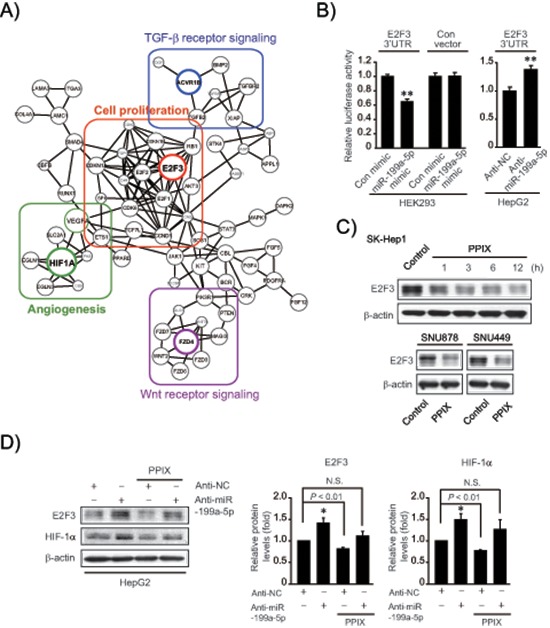
Inhibition of *de novo* synthesis of E2F3 by either miR-199a-5p or PPIX treatment **(A)** An integrative network using putative targets of miR-199a-5p, -519d, and -20b affected by PPIX. Sub-networks were grouped and each subset core gene was bordered and colored. **(B)** E2F3 3′-UTR luciferase assays. Indicated cells were transfected with control mimic (or anti-NC) or miR-199a-5p mimic (or anti-miR-199a-5p) in combination with E2F3 3′-UTR reporter construct. **(C)** Inhibition of E2F3 expression by PPIX treatment. Immunoblottings were done on the lysates of SK-Hep1 cells treated with 3 μM PPIX for the indicated times (upper). SNU878 or SNU449 cells were incubated with 3 μM PPIX for 3 h (lower). **(D)** The effect of anti-miR-199a-5p transfection on E2F3 and HIF-1α expression. HepG2 cells were treated with 3 μM PPIX for 3 h after transfection with anti-NC (control) or anti-miR-199a-5p for 72 h (left). The band intensities of E2F3 or HIF-1α relative to β-actin were quantified by scanning densitometry of the immunoblots (right). The data represent the mean ± S.E. from 3 different independent experiments (significantly different from vehicle-treated control, *P < 0.05; N.S., not significant).

Given the effect of PPIX on miR-199a-5p expression along with its dysregulation in HCC, we next explored the novel target(s) of miR-199a-5p to find the underlying basis of PPIX's anti-cancer effect. First, we created an integrative network using putative targets of the miRNAs affected by PPIX (i.e., miR-199a-5p, -519d, and -20b) because combinatorial regulation is a feature of miRNA regulation and a given miRNA may have multiple targets in similar signaling pathways (Figure 3A). In this approach, we focused on 25 candidate genes that have the potential to be controlled by miR-199a-5p in association with cancer growth or EMT. Forty three genes were additionally chosen as putative targets of miR-519d and -20b. In the integrated analysis, E2F3, HIF1-α, ACVR1B, and FZD4 were linked as key molecules to cell proliferation, angiogenesis, TGF-β signaling, and Wnt signaling sub-networks, respectively (Figure 3A). Specifically, E2F3 was a key bridging molecule to the cell proliferation network.

In subsequent experiments, we determined the ability of miR-199a-5p to inhibit E2F3 using Luc-E2F3 3′-UTR reporter construct, and found that miR-199a-5p mimic transfection inhibited *de novo* synthesis of E2F3 by directly binding to the 3′-UTR of its mRNA; miR-199a-5p mimic decreased luciferase expression from Luc-E2F3 3′-UTR in HEK293 cells, whereas scrambled control miRNA transfection had no effect (Figure 3B). Consistently, treatment with anti-miR-199a-5p significantly enhanced luciferase activity in HepG2 cells, confirming the ability of miR-199a-5p to inhibit E2F3. In addition, treatment of SK-Hep1 cells with PPIX notably diminished E2F3 levels, beginning from 1 h up to 12 h (Figure 3C upper). PPIX inhibition of E2F3 was verified in other mesenchymal tumor cell lines (Figure 3C lower). Treatment of HepG2 cells with anti-miR-199a-5p diminished the inhibitory effects of PPIX on E2F3 and HIF-1α (Figure 3D), supporting the role of PPIX in E2F3 and HIF-1α inhibition, as mediated by increase of miR-199a-5p.

### Upregulation of E2F3 in human HCC samples and in an animal model

**Figure 4:**
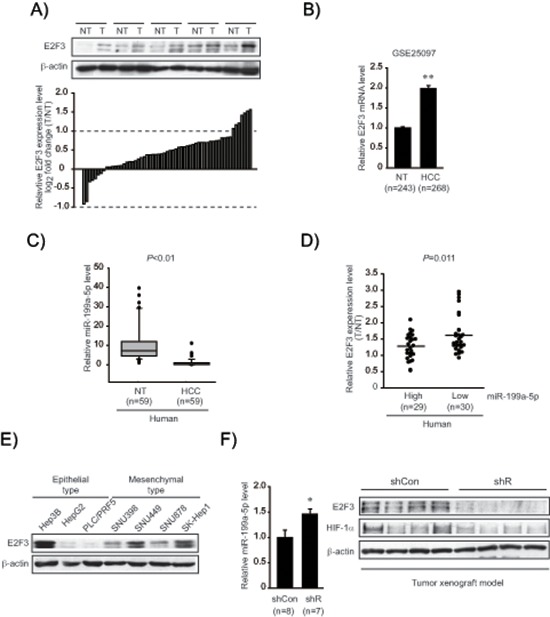
Identification of E2F3 as a novel target of miR-199a-5p **(A)** E2F3 protein levels in human HCC specimens. Immunoblottings for E2F3 were done on 59 pairs of primary HCCs (T) and their adjacent non-tumor liver tissues (NT). A log_2_ fold change more than 1 or less than −1 was considered overexpression or suppression. Equal loading of proteins was confirmed by immunoblottings for β-actin. **(B)** Relative E2F3 mRNA levels in a large cohort of HCC GEO database. Data represents the mean ± S.E. (significantly different from NT, **P < 0.01). **(C)** Relative miR-199a-5p levels in human HCC specimens (*n* = 59, in each group). **(D)** Inverse relationship between E2F3 expression and miR-199a-5p levels in HCC and adjacent non-tumor tissues. The levels of E2F3 were separated into miR-199a-5p high and low expression by the median value. Symbols represent individual samples. **(E)** Immunoblottings for E2F3 in epithelial and mesenchymal liver tumor cell lines. **(F)** Relative miR-199a-5p and E2F3 protein levels in mesenchymal tumor xenografts. The tumors formed in each group at 10 weeks, miR-199a-5p levels were measured using qRT-PCR assays (left). The data represent the mean ± S.E. (significantly different from shCon, *P < 0.05). Representative immunoblottings for E2F3 and HIF-1α are shown (right). shR represents Gα_12_-depletion using a shRNA approach in a SK-Hep1-xenograft model.

To understand biological relevance of the identified target in clinical situations, E2F3 levels were compared in the samples obtained from a group of HCC patients (Figure 4A). E2F3 protein levels were significantly upregulated in a large fraction of human tumor specimens (51 out of 59 sets of paired HCC and adjacent non-tumor samples). In the existing GEO database (GSE25097), E2F3 mRNA levels were also greater in HCC than in adjacent non-tumor liver tissues (Figure 4B). In an effort to confirm a clinical relevance of miR-199a-5p dysregulation and E2F3 upregulation, we measured miR-199a-5p expression in human samples (*n* = 59, in each group); miR-199a-5p levels were significantly lower in HCC specimens than in adjacent non-tumor liver tissues, indicative of miR-199a-5p dysregulation in tumor samples (Figure 4C). Moreover, E2F3 was upregulated when miR-199a-5p level was low in the HCC samples (Figure 4D). To assess whether E2F3 levels were higher in an aggressive type of cancer, we comparatively evaluated the protein levels in a set of epithelial and mesenchymal liver tumor cells. E2F3 levels were greater in all of the classified mesenchymal tumor cell lines compared with the epithelial ones (Figure 4E) (The basal E2F3 levels were inconsistent in Hep3B). As a continuing effort to find the link between EMT and E2F3 overexpression in liver tumor, we took an advantage of the ability of Gα_12_ to promote EMT of liver tumors [27]. Interestingly, Gα_12_-depletion using a shRNA approach (shR) in a SK-Hep1-xenograft model significantly increased miR-199a-5p level (Figure 4F). Immunoblottings verified decreases of E2F3 and HIF-1α in shR xenograft tissues, showing that an approach modulating EMT provided the expected changes in both miR-199a-5p level and E2F3 expression *in vivo*.

### Inhibition of mesenchymal tumor cell growth and migration/invasion

**Figure 5:**
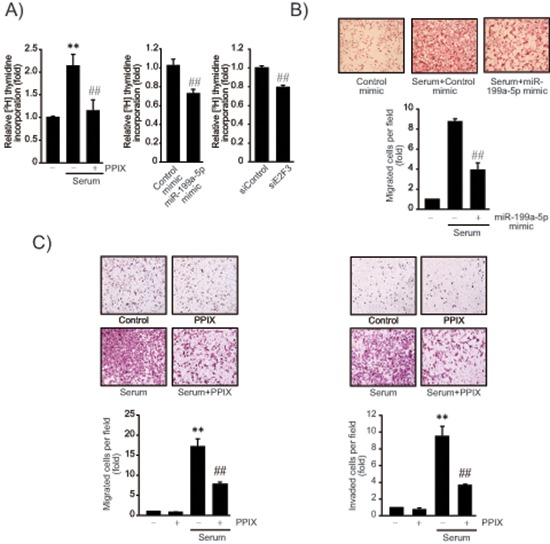
The effects of PPIX on cell proliferation and migration/invasion **(A)** Inhibition of DNA synthesis by PPIX treatment, miR-199a-5p mimic or siE2F3 transfection. The rate of SK-Hep1 cell proliferation was measured using the [methyl-^3^H]-thymidine incorporation assay. DNA synthesis rate was determined in SK-Hep1 cells treated with 3 μM PPIX for 16 h in the presence or absence of 5% fetal bovine serum. The cells were also transfected with control mimic (or siRNA), miR-199a-5p mimic (48 h), or siE2F3 (72 h), and were continuously incubated in a medium containing 5% serum for 16 h. **(B)** Inhibition of cell migration by miR-199a-5p. SK-Hep1 cells were transfected with control miR or miR-199a-5p mimic for 48 h, and were subjected to transwell migration assays in a medium with or without serum for 16 h. **(C)** Inhibition of cell migration and invasion by PPIX. Migrated/invaded cells were examined using light microscopy (magnification, × 200, upper). Numbers of migrate/invaded cells per field were counted and quantified (lower). For A–C, value represents the mean ± S.E. from 3 independent experiments (treatment mean significantly different from vehicle-treated control, **P < 0.01, or serum, ##*P* < 0.01).

Given the role of E2F3 in the promotion of cancer cell proliferation, we next determined the effect of PPIX on DNA synthesis using [^3^H]-thymidine incorporation assay. PPIX treatment inhibited the serum-inducible rate of DNA synthesis in SK-Hep1 cells (Figure 5A, left). Consistently, miR-199a-5p mimic transfection (48 h) significantly diminished tumor cell proliferation (Figure 5A, middle). Knockdown of E2F3 (siE2F3) also suppressed the rate of DNA synthesis (Figure 5A, right), suggesting that E2F3 attribute at least in part to cancer cell proliferation. Thus, the ability of PPIX to inhibit cancer cell may be associated with E2F3 suppression by miR-199a-5p. In addition, transfection with miR-199a-5p mimic significantly inhibited migration of SK-Hep1 cells (Figure 5B). Furthermore, we verified the ability of PPIX to inhibit serum-induced tumor cell migration and invasion (Figure 5C). Together, these results show that increase of miR-199a-5p contributes to the inhibition of tumor cell growth and migration, which may account for the anti-tumor effect of PPIX.

### The effects of PPIX on mesenchymal tumor xenograft model

**Figure 6:**
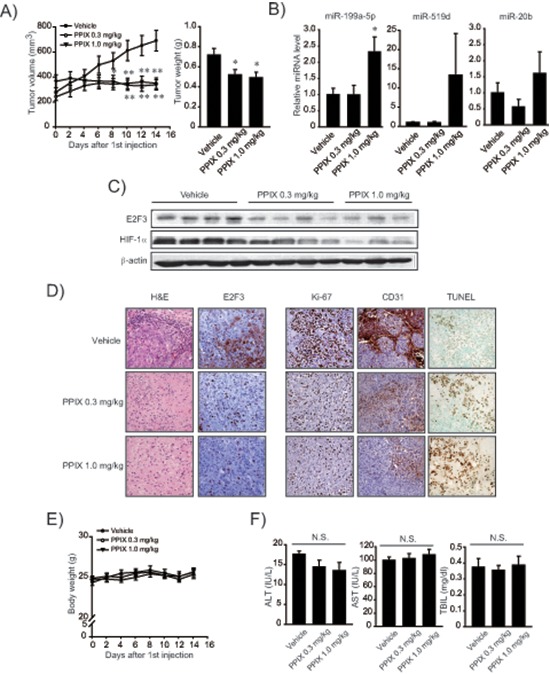
Inhibition of xenograft tumor growth by PPIX **(A)** Inhibition of tumor growth. Volumes of tumors originated from SK-Hep1 were measured every other day with vehicle or PPIX treatment as described in the methods section. The mice were sacrificed on day 14 after PPIX treatment, and the excised tumors were weighed. Value represents the mean ± S.E. (*n* = 8 or 9 in each group, significantly different from vehicle treatment group, *P < 0.05, **P < 0.01, Student's *t*-test). **(B)** qRT-PCR assays for miR-199a-5p, -519d, and -20b in xenograft tumors. Tumors obtained as described above were subjected to analyses. Data represents the mean ± S.E (treatment mean significantly different from vehicle-treated control, *P < 0.05). **(C)** Representative immunoblottings for E2F3 and HIF-1α in the tumor samples. **(D)** Representative hematoxylin and eosin (H&E) staining, immunohistochemistry for E2F3, Ki-67 and CD31, and TUNEL assays (×200, representative figures were shown; *n* = 4 in each group). **(E)** Body weight curves after PPIX treatment at the dose of 0.3 or 1.0 mg/kg body weight for every other day. **(F)** Alanine aminotransferase (ALT), aspartate aminotransferase (AST) activities, and total bilirubin content (TBIL) in serum after PPIX treatments (N.S., not significant).

To understand the miR-199a-5p-mediated anti-cancer effect of PPIX more in depth, we used a mesenchymal tumor xenograft animal model derived from SK-Hep1. Approximately 90% of BALB/c nude mice formed visible and palpable tumor mass two weeks after an injection of SK-Hep1 cells (*n* = 8 or 9, in each group). PPIX treatment (0.3 or 1.0 mg/kg body weight, P.O. every other day for two weeks) significantly reduced the overall tumor growth rate and tumor weight (Figure 6A). We verified a significant increase of miR-199a-5p in the tumor specimens after treatments with PPIX (1.0 mg/kg) (Figure 6B). PPIX treatment also tended to increase miR-519d and -20b levels. Immunoblottings confirmed decreases in E2F3 and HIF-1α levels after PPIX treatments (Figure 6C). Moreover, PPIX at the dose of 0.3 or 1.0 mg/kg suppressed the induction of E2F3, Ki-67, and CD31 in the xenograft tumors (Figure 6D), verifying the ability of PPIX to inhibit tumor cell proliferation and angiogenesis. With the repression of molecular markers, PPIX facilitated tumor cell death, as shown by increased intensities of TUNEL staining (Figure 6D). The lack of changes in body weight, serum alanine aminotransferase, aspartate aminotransferase activities, and total bilirubin content supported that PPIX treatments had no deleterious effect on liver function (Figure 6E and 6F). These results provide an evidence that PPIX effectively inhibits the growth and angiogenic advantage of mesenchymal liver tumor cell as a consequence of the increase of miR-199a-5p targeting E2F3 and HIF-1α.

## DISCUSSION

Hepatocellular carcinoma (HCC) is one of the most prevalent cancers in the world and is classified as a poor prognostic tumor [25]. The high mortality rate of HCC patients is mainly associated with late diagnosis, tumor metastasis, and recurrence after surgical resection [28]. Promising therapy for HCC metastasis is currently very limited. Moreover, EMT phenotype represents chemoresistance, metastasis, and poor prognosis in HCC. Identification of chemicals that have the ability to specifically inhibit the targets responsible for tumor aggressiveness would benefit from HCC therapy [28]. Our results demonstrate that PPIX treatment inhibited the expression of vimentin, Zeb1/2, snail, slug, and twist, EMT markers upregulated in mesenchymal HCC cells, supportive of its anti-cancer potential against HCC with EMT phenotype. Consistently, PPIX exhibited a potent anti-cancer activity in the mesenchymal liver tumor cell.

Unfortunately, liver cancers are resistant to most of chemotherapeutic agents and a promising therapy for HCC metastasis is yet unavailable. Although cancer cells display chemoresistance against doxorubicin and cisplatin [29–31], several studies have reported regimens using the drugs as an alternative therapy of HCC [31, 32]. It has been shown that HIF-1α and P-glycoprotein levels are positively correlated with each other and their overexpression facilitates multidrug resistance in cancer [33]. An important aspect of our study is that PPIX treatment synergistically enhanced the anti-cancer effect of doxorubicin or cisplatin in a mesenchymal tumor cell line. Additionally, we found that PPIX treatment inhibited P-glycoprotein in a time-dependent manner (Supplementary Figure 1). Since the uses of doxorubicin or cisplatin have been limited due to lack of efficacy and dose-related adverse events including cardiomyopathy, nephrotoxicity, and myelosuppression [34, 35], our results raised potential of PPIX as an adjuvant for anti-cancer therapy. It is expected that therapeutically applicable doses of the anti-cancer agents may be reduced when applied in combination with PPIX, to avoid serious adverse effects of chemotherapy. Previously, we showed that hemin, a derivative of PPIX, inhibits HIF-1α and restrains neo-angiogenesis [36]. High levels of PPIX may cause apoptosis of cancer cells in p53-dependent or -independent pathways [37]. However, it remains obscure how certain porphyrins inhibit cell growth and cancer progression. Since PPIX treatment inhibits the expression of major EMT markers increased in mesenchymal cells, the target of PPIX is likely to be associated with repression of the proteins.

Mature miRNAs bind imprecisely with the 3′-UTR regions of complementary target mRNAs and manage diverse enzymatic and regulatory functions. TargetScan 6.1 and miRanda computer algorithms enabled us to select the putative miRNAs interacting with the 3′-UTR of HIF-1α mRNA, and discover the unexpected ability of PPIX to increase miR-199a-5p, -519d, and -20b. Among them, miR-199a-5p is one of the most abundant miRNAs in the liver. miR-199a-5p and miR-199a-3p are both processed from the same precursor, and their levels are lessened in several human cancers including HCC [34, 35]. Cisplatin treatment decreases miR-199a-5p, which may account for cancer chemoresistance in advanced HCC in association with autophagy activation [20]. Consistently, it has been shown that delivery of miR-199a-5p sensitizes cancer cells to doxorubicin [18, 26]. In our previous study, metabolite of oltipraz (M2) was found to elevate miR-199a-5p and -20a/b levels, leading to the inhibition of *HIF1A* translation in colon cancer cells [38]. Here, we report the much greater effect of PPIX on miR-199a-5p (i.e., ~20 fold increase). Our findings showing a substantial increase of miR-199a-5p by PPIX and the link between miR-199a-5p dysregulation and HCC may explain the effectiveness of this agent in mesenchymal HCC, substantiating the therapeutic potential of PPIX as a chemosensitizing agent. p53 may interact with Drosha and facilitate the processing of primary miRNA to precursor form [39]. Since PPIX causes cancer cell apoptosis by interacting with p53 [37], we speculate that p53 may be a regulator of pri-miR-199a-5p processing.

An important finding of this study is the identification of E2F3 as a new target of miR-199a-5p and of the inhibitory effect of PPIX on E2F3. Interestingly, all of the miRNA candidates, except miR-18a/b, targeting HIF-1α have the same putative binding sites in the 3′-UTR region of E2F3 mRNA. Thus, HIF-1α and E2F3 may share common roles in the progression of cancers through the miRNAs identified. The E2F transcriptional factors are downstream targets of the firstly identified retinoblastoma gene (a tumor suppressor), playing a role in cell survival, death, and differentiation [14]. Of eight E2F members, E2F1–3 isoforms work as transcriptional activators, whereas E2F4–8 isoforms act as transcriptional repressors [40]. In particular, E2F3a, a major form of E2F3 expressed in quiescent cells, plays a role in regulating G1/S transition, facilitating tumor growth and apoptosis [21, 41]. In the current study, we found E2F3 overexpression with decrease of miR-199a-5p in mesenchymal liver tumors. Thus, inhibition of E2F3 by PPIX through miR-199a-5p may contribute to suppressing mesenchymal liver tumor growth advantage. Other miRNAs such as miR-20a, -34a, -125b, -200b, -217, and -503 may also suppress E2F3 and induce cancer cell apoptosis [42–44].

PPIX treatment elicits cancer cell apoptosis through its interaction with HSP90, causing p53 accumulation in cells by interrupting the binding between p53 and human double minute 2 [37]. While p53 represses HIF-1α transcriptional activity via p300 [45], HIF-1α stabilizes wild-type p53 [46]. Thus, the inhibitory effect of PPIX on HIF-1α could result from the stabilization of p53. Moreover, their interaction becomes more complicated because p53 and HIF-1α are simultaneously activated in several stress conditions [23, 45, 46]. Consistently, HIF-1α knockdown partially inhibited the proliferation of HCT116, an epithelial cancer cell line, in our previous experiment [36]. HCC cells frequently harbor mutated forms of p53 (i.e., null or minimal p53 activity) [47]. In our study, PPIX treatment inhibited HIF-1α in mesenchymal cells having mutated forms of p53 (SNU398 and SNU449), implying that HIF-1α inhibition by PPIX may not solely depend on p53 accumulation.

PPIX distributes reasonably well within tissues and is metabolized quickly in normal tissue [8]. According to a study describing sono-dynamically induced anti-tumor effect of PPIX on hepatoma-22, PPIX in the plasma decreased at an early time presumably due to its rapid distribution, and the concentration maintained from 4 h to 72 h [48]. In a preliminary experiment, treatment with 0.3 or 1.0 mg/kg PPIX resulted in a comparable effect on E2F3, whereas 0.1 mg/kg PPIX had a mild effect. Since PPIX began to inhibit HIF-1α from the dose of 0.3 mg/kg, we chose 0.3 and 1.0 mg/kg in the xenograft experiment. Our results indicate a saturating and threshold effect of PPIX on E2F3 at the dose of 0.3 mg/kg, showing that PPIX sensitivity may be higher to E2F3 than HIF-1α. The effectiveness of PPIX at the relatively low dose on tumor was also verified by the outcomes of TUNEL, Ki-67 and CD31 assays as well as qRT-PCR for miR-199a-5p. CD31 is basally undetectable in SK-Hep1 despite its endothelial origin [49–51]. Thus, increase of CD31 in the xenograft assay represents angiogenic effect [52]. Overall, our findings support the conclusion that PPIX inhibits mesenchymal tumor growth and angiogenesis, which may depend on the increase of miR-199a-5p.

The synergistic increase in the anti-cancer efficacy of doxorubicin or cisplatin by PPIX in mesenchymal liver tumor cells shown in our data indicates that PPIX may be utilized for the treatment of aggressive HCC. This is supported by the ability of PPIX to increase a specific miRNA involved in the chemoresistance. Moreover, PPIX inhibited E2F3 as well as HIF-1α overexpressed in mesenchymal liver tumor cells. Thus, PPIX may be utilized as an adjuvant of cancer therapy to not only enhance chemosensitivity, but promote its anti-tumor and anti-angiogenesis activities. Our study opens up the possibility of PPIX as a potential candidate in the treatment of advanced HCC.

## MATERIALS AND METHODS

### Materials

PPIX was purchased from Frontier Scientific, Inc (Logan, UT, USA). 3-(4,5-Dimethylthiazol-2-yl)-2,5-diphenyl-tetrazolium bromide (MTT), doxorubicin, cisplatin, and anti-β-actin antibody were provided by Sigma-Aldrich (St. Louis, MO, USA). Antibodies specifically directed against HIF-1α, HIF-1β, and E-cadherin were supplied by BD Biosciences Pharmingen (San Jose, CA, USA). P-Glycoprotein was purchased from Millipore (Billerica, MA, USA). Antibodies recognizing E2F3, Zeb1, Zeb2, vimentin, and twist were obtained from Santa Cruz Biotechnology (Santa Cruz, CA, USA), whereas anti-Snail and anti-Slug antibodies were from Cell Signaling Technology (Beverly, MA, USA). Hsa-miR-199a-5p hairpin inhibitor was purchased from Thermo Scientific (Fremont, CA, USA), whereas scrambled control siRNA and siRNA specifically directed against E2F3 were provided from Dharmacon (Lafayette, CO, USA).

### Cells and cell culture conditions

Human liver tumor cell lines (Hep3B, PLC/PRF5, SK-Hep1, SNU398, SNU449, and SNU878) were obtained from the Korean Cell Line Bank (KCLB) (Seoul, Korea). HepG2 cell line was supplied from the American Type Culture Collection (Manassas, VA, USA). Hep3B, SK-Hep1, and HepG2 cells were maintained in a growth medium containing Dulbecco's modified Eagle's medium (DMEM), 10% fetal bovine serum (FBS), and 1% penicillin-streptomycin whereas PLC/PRF5, SNU398, SNU449, and SNU878 cells were cultured in RPMI-1640 media (Gibco, Gaithersburg, USA) containing 10% FBS and 1% penicillin-streptomycin at 37°C in a humidified atmosphere containing 5% CO_2_. For all experiments, cells were grown to 80–90% confluence at passages between 10 and 30, and were deprived of serum for 16 h before PPIX treatment.

### Immunoblotting assay

Cell lysates were prepared as previously described [36]. Equal loading of proteins was confirmed by immunoblotting for HIF-1β or β-actin. Scanning densitometry of the immunoblots was done using Adobe Photoshop (Photoshop CS6, San Jose, CA, USA).

### MTT assay

MTT assays were carried out according to our previously published paper [53]. Cytotoxicity was defined by the change relative to untreated control.

### Real-time PCR assay

Total RNAs were isolated with Trizol (Invitrogen, Carlsbad, CA, USA) and qRT-PCR assays for mRNAs were performed using LightCycler^®^ DNA master SYBR Green-I kit (Roche, Mannheim, Germany). miRNAs levels were measured using miScript SYBR Green PCR kit (Qiagen) according to the manufacturer's instruction.

### Bioinformatic analyses

Gene targets of miR-199a-5p, -519d, and -20b with conserved seed-match were predicted by TargetScan 6.1 algorithm. Statistically enriched signaling pathways of gene targets of miRNAs were ranked and categorized according to the KEGG pathway using DAVID software. The potential target genes of miRNAs of the most enriched pathway in cancer were extracted. The gene interaction network between the extracted genes was achieved according to the STRING v9.1 database and visualized by Cytoscape software.

### Transfection of miRNAs

The cells were transfected with 100 pmol double-stranded miR-199a-5p mimic (Bioneer, Daejeon, South Korea) or 100 pmol of miR-199a-5p hairpin inhibitor (Dharmacon, Lafayette, CO, USA) with respective negative control using FuGENE^®^ HD Reagent (Roche, Indianapolis, IN, USA). Transfection efficiency was assessed by immunoblotting or qRT-PCR assays. The nucleotide sequences for miR-199a-5p mimic are: 5′-CCCAGUGUUCAGACUACCUGUUC-3′ (guide) and 5′-ACAGGUAGUCUGAACACUGGGUA-3′ (passenger).

### 3′-UTR reporter assay

The plasmid containing Luc-E2F3 3′-UTR (Product ID: HmiT004527, GeneCopoeia, Rockville, MD, USA) was used in reporter assays.

### Patient samples

Fifty nine paired samples of HCC tumor and surrounding normal liver tissues (NT) collected from 2006 to 2009 were supplied by the Asan Medical Center (Seoul, Korea) after institutional review board approval (#2012–0133) in accordance with the ethical guidelines of the 1975 Declaration of Helsinki.

### [^3^H]-Thymidine incorporation assay

[^3^H]-Thymidine incorporation assay was performed as previously described [36].

### *In vitro* cell migration/invasion assays

An *in vitro* cell migration assay was done using a 24-well Transwell^®^ as described previously [36]. Six visual fields were counted for each filter and each sample was assayed in triplicate.

### Xenograft mouse model

Animal studies were conducted in accordance with the institutional guidelines for care and use of laboratory animals. To generate a xenograft tumor model, SK-Hep1 cells (1 × 10^7^ cells) were subcutaneously injected into the left flank of mice (*n* = 10, in each group). Mice with a tumor volume >150 mm^3^ were selected two weeks after the injection and randomly divided into three groups (*n* = 8 or 9, in each group). PPIX (0.3 or 1.0 mg/kg body weight) dissolved in 40% polyethylene glycol 400 was orally administered to the mice every other day for 2 weeks. In a xenograft tumor model, shCon or shR (Gα_12_)-SK-Hep1 (1 × 10^7^ cells) were subcutaneously injected into the left flank of mice (*n* = 10, in each group). Tumor volumes were calculated using the following formula: tumor volume (cm^3^) = 0.52 × (width)^2^ × (length).

### Immunohistochemistry

Tumor tissues were fixed in 10% formalin, embedded in paraffin, and the samples were cut by a microtome into 4 μm thick sections, and mounted on slides. Tissue sections were immunostained with the antibody directed against E2F3 (EMD Millipore, Billerica, MA, USA), Ki-67 (Diagnostic BioSystems, Pleasanton, CA, USA) or CD31 (Thermo Scientific, Fremont, CA, USA).

### Statistical analysis

Statistical significance was assessed using SPSS 20.0 by one-way analysis of variance procedures and unpaired or paired Student's *t*-test.

## SUPPLEMENTARY FIGURE



## Abbreviations

EMT: epithelial-mesenchymal transition
E2F3: E2F transcription factor 3
HCC: hepatocellular carcinoma
HIF: hypoxia inducible factor
miRNA: microRNA
MTT: 3-(4,5-dimethylthiazol-2-yl)-2,5-diphenyl-tetrazolium bromide
PPIX: protoporphyrin IX
UTR: untranslated region
