# Supplementary material for: Increase of miR-199a-5p by protoporphyrin IX, a photocatalyzer, directly inhibits E2F3, sensitizing mesenchymal tumor cells to anti-cancer agents
Source: Oncotarget. 2015 Feb 24;6(6):3918–31. doi: 10.18632/oncotarget.2928 (PMC4414163; doi:10.18632/oncotarget.2928)
Supplement: Supplementary file 1 [file oncotarget-06-3918-s001.pdf]

SUPPLEMENTARY FIGURE

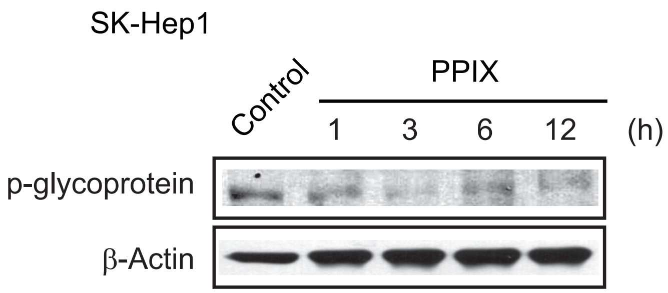

Supplementary Figure 1: Immunoblotting for p-glycoprotein in SK-Hep1 cells treated with vehicle or 3 μM PPIX for 1–12 h.
